# Optomechanical crystal with bound states in the continuum

**DOI:** 10.1038/s41467-022-30965-6

**Published:** 2022-06-08

**Authors:** Shengyan Liu, Hao Tong, Kejie Fang

**Affiliations:** 1 Holonyak Micro and Nanotechnology Laboratory and Department of Electrical and Computer Engineering, University of Illinois at Urbana-Champaign, Urbana, IL 61801 USA; 2 Illinois Quantum Information Science and Technology Center, University of Illinois at Urbana-Champaign, Urbana, IL 61801 USA

**Keywords:** Photoacoustics, Optomechanics, Photonic crystals

## Abstract

Chipscale micro- and nano-optomechanical systems, hinging on the intangible radiation-pressure force, have shown their unique strength in sensing, signal transduction, and exploration of quantum physics with mechanical resonators. Optomechanical crystals, as one of the leading device platforms, enable simultaneous molding of the band structure of optical photons and microwave phonons with strong optomechanical coupling. Here, we demonstrate a new breed of optomechanical crystals in two-dimensional slab-on-substrate structures empowered by mechanical bound states in the continuum (BICs) at 8 GHz. We show symmetry-induced BIC emergence with optomechanical couplings up to *g*/2*π* ≈ 2.5 MHz per unit cell, on par with low-dimensional optomechanical crystals. Our work paves the way towards exploration of photon-phonon interaction beyond suspended microcavities, which might lead to new applications of optomechanics from phonon sensing to quantum transduction.

## Abstract (web summary)

Unsuspended optomechanical systems might enable unique applications for quantum sensing and transduction. Here, the authors demonstrate a two-dimensional slab-on-substrate optomechanical crystal empowered by mechanical bound states in the continuum.

## Introduction

Cavity-optomechanics has attracted extensive studies in recent years because of the rich physics associated with the nonlinear optomechanical interaction and a broad range of prospective applications from signal transduction to sensing^1^. One of the leading optomechanical device architectures is optomechanical crystals^2^, where micro- and nano-scale structures give rise to strong radiation-pressure force coupling between wavelength-similar optical photons and microwave phonons. By band-structure engineering of suspended optomechanical crystals, both one-dimensional and quasi-two-dimensional defect cavities^2–4^ have been created with long-lived optical and mechanical resonances^5^. Such optomechanical crystal microcavities have enabled groundbreaking quantum experiments including ground-state cooling of mechanical resonators^6^, testing Bell-type inequalities^7^, and a mechanical quantum memory^8^.

Despite the success of optomechanical microcavities, it is highly desirable to explore two-dimensional optomechanical crystals. On one hand, two-dimensional optomechanical crystals offer more degrees of freedom for manipulation of photon-phonon interaction to induce collective phenomena^9–12^. On the other hand, extended optomechanical crystals, especially in unsuspended structures^13–15^, might alleviate the optical-absorption-induced heating that plagues released microcavities^16^. Ideally, such slab-on-substrate optomechanical crystals should facilitate the dissipation of heat phonons while sustaining long-lived mechanical resonances in the device layer.

Recently, mechanical bound states in the continuum (BICs) are observed in two-dimensional slab-on-substrate phononic crystals^17^. Despite having a zero Bloch wavevector and thus spectrally immersing in the sound cone of the substrate, these mechanical BICs are confined in the slab because of the symmetry-induced decoupling from the acoustic radiation field. There are also proposals and demonstrations of mechanical BICs in microcavities due to, for example, accidental radiation amplitude cancellation^18,19^. A significant step forward thus would be coupling mechanical BICs with optical resonances in an optomechanical crystal which will bring the effective radiation-pressure force control and associated functionalities.

In this work, we realize two-dimensional silicon-on-insulator optomechanical crystals with mechanical BICs coupled with optical guided resonances. In such periodic optomechanical crystals, the radiation-pressure coupling between the mechanical BIC and optical modes strongly depends on the mode symmetry^20^, which in many cases dictates an adversely null optomechanical coupling. Here, taking into account both symmetry of the optomechanical crystal and the silicon crystal lattice, we are able to achieve optomechanical coupling up to *g*/2*π* ≈ 2.5 MHz per unit cell between a mechanical BIC at 8 GHz and an optical band-edge mode at 193 THz, which is comparable to one-dimensional suspended optomechanical crystals^21^. With optically-transduced mechanical spectroscopy at room temperature, we demonstrate control of mechanical BICs and optomechanical coupling via the interplay of symmetry of the optomechanical crystal and crystalline material. Our work paves the way for the study of photon-phonon interaction in BIC optomechanical crystals at low temperatures, when the benefit of slab-on-substrate device architecture is expected to arise, and Floquet topological physics beyond the tight-binding model^22^.

## Results

### Design of BIC optomechanical crystals

**Fig. 1:**
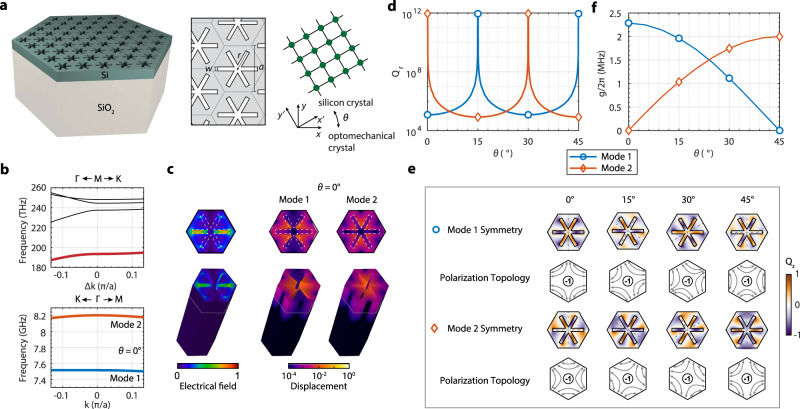
Two-dimensional optomechanical crystal with mechanical BICs. **a** A schematic diagram of the two-dimensional silicon-on-insulator optomechanical crystal and its unit-cell structure. The optomechanical crystal could be rotated by an angle *θ* relative to the silicon crystal lattice. **b** Optical band structure near the *M* point (top) and mechanical band structure near the Γ point (bottom) for *θ* = 0. The relevant optical and mechanical bands are highlighted in color. **c** Simulated fundamental TE-like optical mode at the *M* point (normalized total electric field) and mechanical modes 1 and 2 at the Γ point (normalized total displacement field). **d** Simulated radiative quality factor of the mechanical modes with respect to *θ*. **e** Mechanical mode symmetry is illustrated using the normalized *z*-direction displacement, momentum-space transverse polarization distribution, and the winding number. **f** Unit-cell optomechanical coupling of mechanical modes 1 and 2 with the fundamental TE-like optical mode.

The designed optomechanical crystal in the silicon-on-oxide material system has a hexagonal “snowflake” unit cell (Fig. 1a). The reason behind this design is that sixfold symmetric structures tend to yield mechanical BICs coupled with optical guided resonances^20^ and, also, the spike feature of the “snowflake” could lead to sizable vibrations for large optomechanical couplings. We here consider optical modes at the *M* point below the light cone and mechanical modes at the Γ point. The latter is necessary because mechanical vibrations generally induce a linear perturbation of the optical mode energy, i.e., $\delta U \sim \int \delta x({{{{{\bf{R}}}}}})\varepsilon {{{{{\rm{|}}}}}}E({{{{{\bf{R}}}}}}){{{{{{\rm{|}}}}}}}^{2}d{{{{{\bf{R}}}}}}$, and this integral is nonzero in a periodic structure only when the mechanical displacement *δx* has a zero Bloch wavevector (Supplementary Information (SI)). Since the refractive index of crystalline silicon is isotropic, the symmetry of optical modes at the *M* point is governed by the *C*_2*v*_ group. We select the fundamental transverse-electric(TE)-like mode which is odd with respect to the *xz*-plane. The stiffness tensor of silicon, on the other hand, is anisotropic in the crystal plane with a *C*_4*v*_ group symmetry, which is incommensurable with the *C*_6*v*_ group symmetry of the hexagonal optomechanical crystal. As a result, the symmetry of mechanical modes at the Γ point will depend on the orientation angle *θ* between the optomechanical crystal and the silicon crystal lattice. When *θ* = 0°, 15°, 30°, and 45°, the symmetry group of the mechanical mode is *C*_2*v*_, while for other orientations it will be *C*_2_. Only *C*_2*v*_ group supports mechanical BICs which decouple from both transverse and longitudinal radiation waves^17^, whose displacement field is perpendicular and parallel to the wavevector, respectively.

Figure 1b shows the optical band structure near the *M* point and mechanical band structure near the Γ point for *θ* = 0° and (*r, w, a, t, h*) = (167, 34, 389, 220, 3000) nm, where *t* and *h* are the thickness of the silicon and oxide layers, respectively, *a* is the lattice constant, and (*r,w*) are “snowflake” dimensions. The fundamental TE-like optical mode has a frequency of 193 THz and the relevant mechanical modes have frequencies of about 7.5 and 8.2 GHz, respectively. Their mode profiles are shown in Fig. 1c. Simulation shows that the radiation quality factor *Q*_*r*_, i.e., the ratio between the frequency and radiation loss rate, of mechanical mode 2 (8.2 GHz) diverges and that of mode 1 is finite but remains relatively high compared to other lossy modes. As a result, for *θ* = 0°, modes 2 and 1 are mechanical BIC and quasi-BIC, respectively. The fact that mode 1 is a quasi-BIC is because the two mechanical bands are degenerate at the Γ point when the stiffness tensor is isotropic and both modes are BICs belonging to the *E*_2_ representation of the *C*_6*v*_ group; the actual anisotropic stiffness tensor of silicon splits the degeneracy, reducing the *E*_2_ representation to *A*_2_ (BIC) and *A*_1_ (quasi-BIC) representations of the *C*_2*v*_ group. Mode 1 turns out to have the *A*_1_ representation, leading to coupling with the longitudinal radiation wave. When *θ* changes, BIC and quasi-BIC alternate between modes 1 and 2 (while the frequencies of the two modes remain almost unchanged) as shown in Fig. 1d. This is further illustrated in Fig. 1e using the mode symmetry. Below, the mode symmetry under certain symmetry operations is defined with regard to the vector parity of the electric field of the optical mode or the displacement field of the mechanical mode. Taking *θ* = 0° as an example again, both mode 1 and 2 are even under the 180° rotation, which leads to decoupling from the transverse radiation wave. In addition, mode 2(1) is odd(even) with respect to the symmetry axes (dashed lines), resulting in decoupling from (coupling with) the longitudinal radiation wave and thus a rigorous(quasi) BIC. Same arguments apply to the other three *θ*s while noticing the mirror symmetry axes rotate with *θ*. The mechanical BIC and quasi-BIC are also associated with transverse topological charges, defined as the winding number of far-field transverse polarization around the Γ point^17^. The polarization fields rotate together with *θ*, which is unique for the anisotropic mechanical system. For orientations other than the four specific angles, the two modes belong to the *A* representation of the *C*_2_ group and thus are quasi-BICs which only couple to the longitudinal acoustic waves. As a result, their quality factor over 10^4^ is significantly higher than unconfined modes.

The interaction between the optical and mechanical modes can be analyzed using mode symmetry (SI). Roughly, because the *M*-point optical mode energy density is even with respect to the *xz*-plane, mechanical modes that are odd with respect to the *xz*-plane, including the BICs for *θ* = 0° and 45°, will not interact with the optical mode. For other cases, the optomechanical coupling could be nonzero (see Table 1 for a summary), thanks to the incommensurable symmetry of the optomechanical crystal and silicon lattice crystal. The bare optomechanical coupling of a unit cell, *g*, including both moving boundary and photoelastic effects, is calculated and plotted in Fig. 1f. For example, the BIC at *θ* = 15° has *g*/2*π* = 1.96 MHz, with a contribution from the moving boundary and photoelastic effect of 0.61 and 1.35 MHz, respectively. The coupling *g* of the “snowflake” optomechanical crystal will increase with smaller air gaps. Here, with a practical air gap w ≈ 30 nm, the BIC optomechanical crystal achieves a coupling rate (per unit cell) on par with one-dimensional suspended optomechanical crystals^21^.

**Table 1:** Summary of mechanical mode representation and optomechanical coupling.

	0°	15°	30°	45°
Mode 1	*A*_1_, quasi-BIC	*A*_2_, BIC	*A*_1_, quasi-BIC	*A*_2_, BIC
*g*	≠0	≠0	≠0	0
Mode 2	*A*_2_, BIC	*A*_1_, quasi-BIC	*A*_2_, BIC	*A*_1_, quasi-BIC
g	0	≠0	≠0	≠0

### Photonic crystal band-edge multimodes

**Fig. 2:**
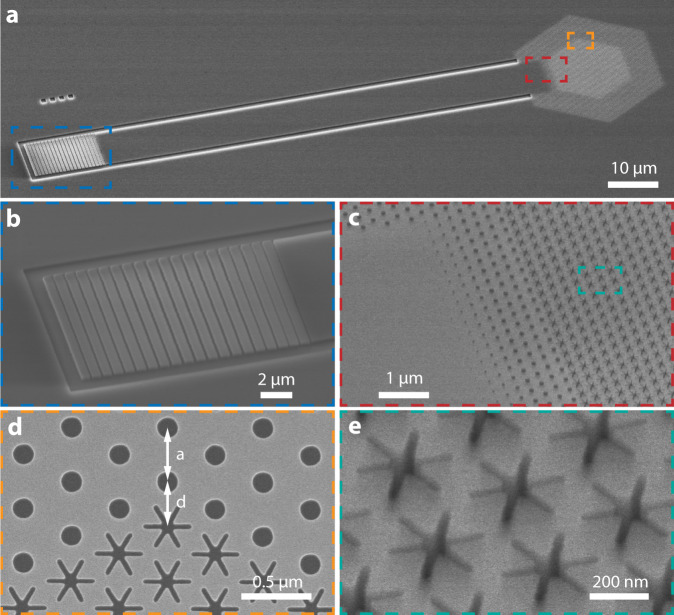
Scanning electron microscopy of the optomechanical crystal device. **a** Full device. **b** Apodized grating coupler. **c** Junction between the optomechanical crystal and waveguide with tapered photonic crystal mirrors. **d** Boundary between the optomechanical crystal and photonic crystal mirror. The photonic crystal mirror is displaced by *d ‒ a*. **e** Zoom-in view of the optomechanical crystal.

The optomechanical crystal device is fabricated from a silicon-on-insulator wafer with a 220 nm thick silicon device layer and 3 μm buried oxide. The air gap of the “snowflake” as small as 30 nm is achieved. The device consists of several functional components (Fig. 2). The hexagonal BIC optomechanical crystal has *N* unit cells along each edge and is surrounded by photonic crystal mirrors with a triangular lattice of cylindrical holes on five edges. The photonic crystal mirror, with the same lattice constant as the snowflake optomechanical crystal, is designed to have a complete TE-like bandgap with the center wavelength around 1550 nm to suppress the lateral radiation of the optical band-edge mode. The photonic crystal mirror is also slightly displaced to minimize the out-of-plane radiation due to the boundary effect on the finite-size optical mode (Fig. 2d). One side of the hexagonal snowflake crystal is connected to a waveguide terminated with an apodized grating coupler for coupling light from a single-mode optical fiber. This configuration makes the optomechanical crystal effectively a one-port device with (mode-dependent) external, i.e., to-waveguide, and intrinsic loss rate of *κ*_*e*_ and *κ*_*i*_, respectively. The former can be controlled by the number of photonic crystal mirror layers between the waveguide and optomechanical crystal (Fig. 2c) to achieve different coupling conditions including the critical coupling, i.e., ${\kappa }_{e}\approx {\kappa }_{i}$, which is desirable for sideband-resolved mechanical spectroscopy.

**Fig. 3:**
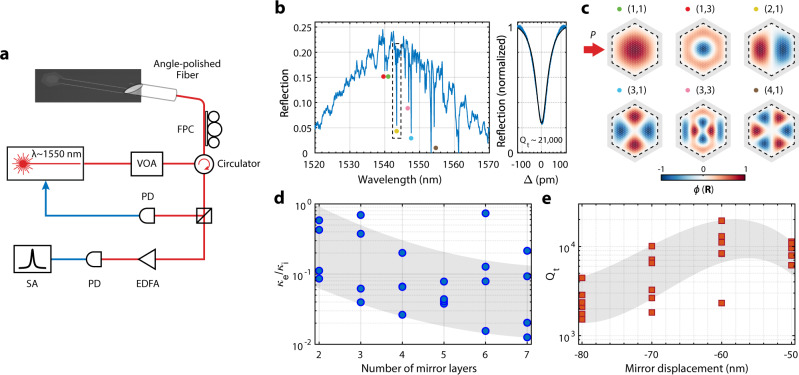
Multimode optical spectroscopy. **a** Schematic diagram of the measurement setup. The pump light is guided into the on-chip device using an angle-polished fiber. The reflected light is divided by a directional coupler for optical (10%) and mechanical (90%) spectroscopy. The optical signal is used to lock the laser-cavity detuning. VOA variable optical attenuator, FPC fiber polarization controller, EDFA erbium-doped fiber amplifier, PD photodetector, SA spectrum analyzer. **b** Optical spectrum of a device with multiple standing-wave resonances, one of which has a total quality factor of 21,000 (right). **c** Mode envelope *ϕ*(**R**) of standing-wave resonances with given orders corresponding to the dotted resonances in **b**. The arrow indicates the waveguide input. **d** ${\kappa }_{e}/{\kappa }_{i}$ for different numbers of nominal mirror layers at the junction between the optomechanical crystal and waveguide. For each number of mirror layers, multiple devices with different photonic crystal mirror displacements are measured. **e** Total optical quality factor versus the displacement of the photonic crystal mirror, i.e., *d* ‒ *a*. **e** is plotted using the same set of data as **d**.

The device is measured using the setup shown in Fig. 3a. An angle-polished optical fiber is used to guide light via the on-chip apodized grating coupler^23,24^ to the optomechanical crystal and collect the reflected light. The angle of the fiber (≈35.5°) and the apodized grating coupler are co-designed to realize an optimized coupling efficiency of 52% for 1550 nm light and 3-dB bandwidth of 40 nm (SI). Because of the reflection at the boundary of finite-size photonic/phononic crystals, band-edge standing-wave resonances will be formed^17,25–28^. The mode envelope of the standing-wave resonances can be approximated by the (*p,q*)-th order eigenfunctions of a flat-top potential well within the boundary of the photonic/phononic crystal^29^. Figure 3b shows the optical reflection spectrum of a hexagonal optomechanical crystal with *N* = 25, where a series of band-edge standing-wave resonances are observed. The major order *p* of a standing-wave resonance is identified from the group of resonances it belongs to and the minor order *q* is determined by the position of the resonance in a group. Because of the hyperbolic paraboloid topology of the band structure near the *M* point, resonances with smaller *p* and larger *q* will have shorter wavelengths. In addition, only *q*-odd resonances are observed because the excited waveguide mode is even with respect to the center of the waveguide. Order *q* = 1 modes are expected to have the deepest resonance dip given they have the largest *κ*_*e*_ close to the critical coupling. The mode envelope *ϕ*(**R**) of some standing-wave resonances are shown in Fig. 3c.

The number of photonic crystal mirror layers at the waveguide-optomechanical crystal junction is varied to tune ${\kappa }_{e}/{\kappa }_{i}$, which is extracted by fitting the optical resonance spectrum using the normalized reflection coefficient of the one-port waveguide-coupled resonator, $R[\omega ]={\left|1-\frac{{\kappa }_{e}}{i(\omega -{\omega }_{o})+({\kappa }_{e}+{\kappa }_{i})/2}\right|}^{2}$. Figure 3d shows ${\kappa }_{e}/{\kappa }_{i}$ of the (1,1) standing-wave resonance of a group of devices, where a decreasing trend over the number of junction mirror layers is observed as a result of the reduced coupling between the waveguide mode and standing-wave resonances. The displacement of the photonic crystal mirror around the optomechanical crystal is also varied to optimize the total quality factor of the standing-wave resonance. As shown in Fig. 3e, a displacement of −60 nm from the nominal lattice constant is optimal, which is consistent with the simulation (SI). We note the variation of photonic crystal mirrors changes not only *κ*_*e*_ but also *κ*_*i*_ via the perturbation of the evanescent field, leading to fluctuations of ${\kappa }_{e}/{\kappa }_{i}$. An optimized (2,1) optical resonance is shown in Fig. 3b with a total quality factor of 21,000 while being close to the critical coupling. The quality factor of the optical resonance and its variation is largely due to the disorder-induced scattering to the modes above the light cone^26^.

### Room temperature mechanical spectroscopy

We perform the mechanical spectroscopy at room temperature using a blue-detuned laser with a frequency ${\omega }_{l}={\omega }_{o}+{\omega }_{m}$, where *ω*_*o*_ and *ω*_*m*_ are the frequencies of the optical and mechanical band-edge modes, respectively. We stabilize the laser-cavity-detuning by locking the device-reflected power with feedback control of the laser frequency (Fig. 3a). For each device, an optical standing-wave resonance with *Q*_*t*_ about 2 × 10^4^ and ${\kappa }_{e}/{\kappa }_{i}$ close to 1 is chosen, which means the device is operated near the sideband-resolved regime, i.e., ${\omega }_{m}\approx {\omega }_{o}/{Q}_{t}$. The reflected pump light with sidebands due to the modulation of mechanical modes is sent to a high-speed photodetector. The beating between the pump and sidebands thus yields the mechanical spectrum which is observed by a spectrum analyzer.

**Fig. 4:**
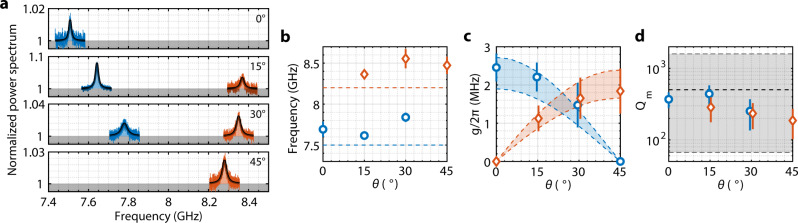
Room temperature mechanical spectroscopy. **a** Measured mechanical noise power spectrum for one device each for *θ* = 0°, 15°, 30°, and 45°. **b** Mechanical frequency distribution of a group of five devices for each *θ*. Error bars indicate one standard deviation (error bars smaller than the symbol are not shown). Dashed lines are the simulated mechanical frequencies. **c** Unit-cell optomechanical coupling. The dashed lines correspond to simulated coupling for *w* = 28 nm (top) and 40 nm (bottom). **d** Total quality factor of the mechanical modes. The dashed lines are the estimated bound and mean from the simulation of a 1 × 4 super-cell with disorders.

Devices with different orientations between the optomechanical crystal and the silicon crystal lattice are fabricated to reveal the mechanical (quasi-)BICs and the impact of symmetry on the optomechanical interaction. Figure 4a shows the mechanical spectroscopy of a group of devices with *θ* = 0°, 15°, 30°, and 45°. The spectrum is normalized with respect to the total background noise. At *θ* = 0° and 45°, only the quasi-BIC mode is observed while the BIC mode is invisible because of the symmetry-inhibited optomechanical coupling. At *θ* = 15° and 30°, both BIC and quasi-BIC modes are detected. These observed mechanical modes are the (1,1) standing-wave resonance, while higher-order resonances are obscure because of both lower quality factors and optomechanical coupling. Figure 4b shows the frequency distribution of a group of devices. The occurrence of the mechanical BIC and quasi-BIC modes and their frequencies are consistent with the simulation.

Under a blue-detuned pump, the optomechanical interaction between the mechanical and optical resonances is described by a linearized two-mode squeezing Hamiltonian, $\hat{H}=G({\hat{a}}^{{{\dagger}} }{\hat{b}}^{{{\dagger}} }+\hat{a}\hat{b})$, where ${\hat{a}}^{{{\dagger}} }$(${\hat{b}}^{{{\dagger}} }$) and $\hat{a}$($\hat{b}$) are the creation and annihilation operators of the optical(mechanical) resonance. The parametrically-enhanced optomechanical mode coupling *G* of standing-wave resonances is given by

1 $$G=\zeta g\sqrt{{n}_{c}},$$

where *n*_*c*_ is the average number of photons in one unit cell and *ζ* is a resonance-dependent *O*(1) parameter due to the finite-mode-size correction (SI). Given the nature of the weak radiation-pressure force, the important coupling in generic optomechanical systems is the parametric mode coupling, which is related to the cooperativity given by $C=4{G}^{2}/\kappa \gamma$. It is seen from Eq. 1 that the parametric mode coupling of a finite optomechanical crystal is primarily determined by the unit-cell coupling and the per unit cell photon number, and is independent of the size of the crystal. In other words, despite that the bare mode coupling of optomechanical crystals roughly scales as 1/*N*, it can be compensated by the large number of photons available which scales with *N*^2^. The per unit cell photon number is largely constrained by the thermo-optic effect and the heat capacity of the unit cell, which the slab-on-substrate structure can ameliorate, especially when the thermal conductivity of the substrate is comparable to the slab. In our experiments, the device is typically operated under a pump power *P* of a few mW and ${n}_{c}={\kappa }_{e}P/({({\omega }_{m})}^{2}+{(\kappa /2)}^{2})/\frac{3\sqrt{3}}{2}{N}^{2}$ is on the order of 10-20. The unit-cell optomechanical coupling *g* is extracted from the noise power spectrum (see Methods and SI) and plotted in Fig. 4c. Large optomechanical coupling *g*/2*π* ≈2.5 MHz is observed for the mechanical BIC. The deviation from the simulated value could be due to the variation of the actual snowflake gap size and disorders in the optomechanical crystal. Despite being unreleased and two-dimensional, the optomechanical interaction (per unit cell) in the BIC optomechanical crystal is on par with the suspended low-dimensional optomechanical crystal devices, such as nanobeams.

The quality factor of the observed mechanical modes is shown in Fig. 4d. There are two main factors limiting the mechanical quality factor of the current device. First, the size of the optomechanical crystal is *N* = 25, corresponding to a finite wavevector of $k\approx 2\pi /(3{Na})$ for the fundamental standing-wave resonance. The radiation quality factor of mechanical standing-wave resonances with finite wavevectors decreases drastically as they deviate from the BIC at the Γ point^17^. Second, the fabricated optomechanical crystal has critical dimensions as small as 30 nm, which cause random variations among unit cells. These disorders induce scattering between different orders of band-edge resonances, which effectively introduces more radiation channels to a given resonance and degrades its quality factor^26,30,31^. The lateral radiation for this size of the crystal is not a dominant loss according to the simulation and can be optimized with a phononic crystal mirror. In addition, at room temperature, the quality factor of GHz-frequency mechanical modes is ultimately limited by material absorption to around 1000–2000^2^. Because of these, there is no significant statistical difference between the quality factor of BIC and quasi-BIC modes. The observed quality factor is verified with numerical simulations of finite super-cells with realistic disorders (SI). However, the mechanical radiation quality factor will increase with the size of optomechanical crystals^17^, which will be critical to low-temperature measurements when the material absorption is suppressed.

## Discussion

In summary, we have realized the first two-dimensional slab-on-substrate optomechanical crystals with mechanical BICs. Guided by a symmetry-based design approach, this architecture offers optomechanical interaction (per unit cell) on par with suspended optomechanical crystal devices. The two-dimensional optomechanical crystal with tunable symmetry provides an arena for the exploration of rich multimode physics^32,33^. In addition, the cavity-less optomechanical crystal might realize Floquet topological physics beyond the tight-binding model^22^. The benefit of the slab-on-substrate device architecture in terms of heat dissipation is obscured at room temperature, especially for optomechanical crystals with a large area-to-perimeter ratio, because of the low thermal conductivity of the oxide substrate two orders smaller than silicon. However, such benefit will become evident at low temperatures (<1 K), relevant for quantum experiments involving GHz mechanical modes, or in a different material system, when/where the thermal conductivity of the substrate and slab becomes comparable^34,35^. Besides the suppressed material-absorption loss at low temperatures, the mechanical quality factor could be enhanced using the merging BIC mechanism^26^ and implementing appropriate phononic crystal mirrors. As a consequence, slab-on-substrate BIC optomechanical crystals with improved optical and mechanical losses could be unique at low temperatures for exploration of modalities including phonon sensing and macroscopic mechanical oscillators in the quantum regime^36,37^.

## Methods

### Device fabrication

Devices are fabricated in silicon-on-insulator microchips (220 nm silicon device layer and 3 μm buried oxide layer) using electron beam lithography with ZEP520A mask, followed by inductively coupled plasma reactive ion etch of silicon using SF_6_ and CHF_3_.

### Mechanical noise power spectrum

The total noise power spectral density measured by the photodetector is given by refs. ^16,38^

2 $$S[\omega ] = {S}_{e}+\frac{{G}_{e}^{2}}{{R}_{I}}\left({S}_{{{{{{\rm{EDFA}}}}}}}+{G}_{{{{{{\rm{EDFA}}}}}}}^{2}{S}_{{{{{{\rm{SN}}}}}}}^{2}(1+\eta \frac{{\kappa }_{e}}{\kappa }\frac{8{G}^{2}}{\kappa }{S}_{m}[\omega ])\right)$$

where *S*_*e*_ is the electronic noise of the detector, *S*_EDFA_ is the noise of EDFA, ${S}_{{{{{{\rm{SN}}}}}}}=\sqrt{2{P}_{{{{{{\rm{out}}}}}}}{\hslash }{\omega }_{l}}$ is the optical shot noise, ${S}_{m}[\omega ]$ is the mechanical noise spectrum, $\kappa ={\kappa }_{i}+{\kappa }_{e}$ is the total loss rate of the optical resonance, *G* is the parametrically-enhanced optomechanical coupling, *η* is the total detection efficiency, *G*_EDFA_ is the EDFA gain, *G*_*e*_ is the detector gain factor from optical power to voltage, and *R*_*I*_ is the input impedance of the spectrum analyzer. The optically-transduced mechanical noise spectrum is given by

3 $${S}_{m}[\omega ]=\frac{1}{2}\left(\frac{\gamma ({\bar{n}}_{m}+1)}{{\left({\omega }_{m}-\omega \right)}^{2}+{\left(\gamma /2\right)}^{2}}+\frac{\gamma {\bar{n}}_{m}}{{\left({\omega }_{m}+\omega \right)}^{2}+{\left(\gamma /2\right)}^{2}}\right)$$

with ${\bar{n}}_{m}=\frac{{k}_{B}T}{{\hslash }{\omega }_{m}}$ the thermal occupation of the mechanical mode. The total detection efficiency is ${\eta} ={\eta}_{{{{{\rm{cpl}}}}}}{\eta}_{{{{{{\rm{t}}}}}}}{\eta}_{{{\det}}}$, where ${\eta }_{{{{{\rm{cpl}}}}}}$ is the coupling efficiency of the grating coupler at the pump wavelength, *η*_*t*_ is the total transmission efficiency in the optical fiber path from the chip to the detector, and *η*_det_ is the quantum efficiency of the photodetector. We measured *η*_t_ = 0.80 and *η*_cpl_ ≈ 0.5 depending on the pump wavelength, while *η*_det_ = 0.68 is given by the photodetector, which results in *η* ≈ 0.27. *S*_*e*_ is determined by blocking the light and $\frac{{G}_{e}^{2}}{{R}_{I}}{G}_{{{{{{\rm{EDFA}}}}}}}^{2}{S}_{{{{{{\rm{SN}}}}}}}^{2}$ is determined by removing EDFA while keeping the optical power incident onto the photodetector the same. Then the measured noise power spectrum is fitted using Eqs. 2 and 3, with *ω*_*m*_, *γ*, and *G* the only fitting parameters. The unit-cell optomechanical coupling is calculated from $g=G/(\zeta \sqrt{{n}_{c}})$, using ${n}_{c}={\kappa }_{e}P/({({\omega }_{m})}^{2}+{(\kappa /2)}^{2})/\frac{3\sqrt{3}}{2}{N}^{2}$ and *ζ* depending on the optical standing-wave resonance that is used for the mechanical spectroscopy (SI).

## Supplementary information

Supplementary Information

## Data Availability

Data supporting the findings of this study are available within the article and its Supplementary Information, or from the corresponding author upon reasonable request.
