# Author Correction: Optomechanical crystal with bound states in the continuum

**DOI:** 10.1038/s41467-022-32666-6

**Published:** 2022-08-19

**Authors:** Shengyan Liu, Hao Tong, Kejie Fang

**Affiliations:** 1 Holonyak Micro and Nanotechnology Laboratory and Department of Electrical and Computer Engineering, University of Illinois at Urbana-Champaign, Urbana, IL 61801 USA; 2 Illinois Quantum Information Science and Technology Center, University of Illinois at Urbana-Champaign, Urbana, IL 61801 USA

**Keywords:** Photoacoustics, Optomechanics, Photonic crystals

Correction to: *Nature Communications* 10.1038/s41467-022-30965-6, published online 08 June 2022

In the Acknowledgements section of this article the grant number relating to U.S. National Science Foundation was incorrectly given as 2016136 and should have been 2137642. The original article has been corrected.

