# Supplementary material for: Optomechanical crystal with bound states in the continuum
Source: Nat Commun. 2022 Jun 8;13:3187. doi: 10.1038/s41467-022-30965-6 (PMC9177575; doi:10.1038/s41467-022-30965-6)
Supplement: Supplementary file 1 — Supplementary Information [file 41467_2022_30965_MOESM1_ESM.pdf]

Supplementary Information for "Optomechanical crystal with bound states in the continuum"

Shengyan Liu,[1,2,*] Hao Tong,[1,2,*] and Kejie Fang[1,2,†]

[1] *Holonyak Micro and Nanotechnology Laboratory and Department of Electrical and Computer Engineering, University of Illinois at Urbana-Champaign, Urbana, IL 61801 USA*

[2] *Illinois Quantum Information Science and Technology Center, University of Illinois at Urbana-Champaign, Urbana, IL 61801 USA*

I. SYMMETRY ANALYSIS OF MECHANICAL BICS AND OPTOMECHANICAL COUPLING

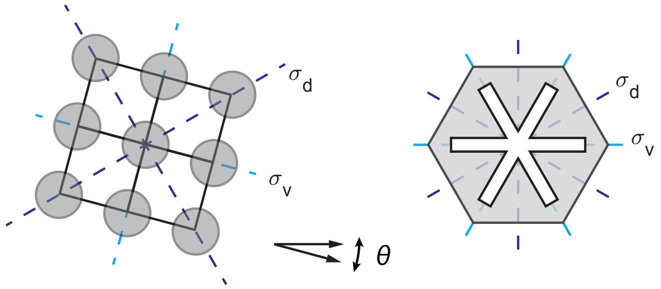

FIG. S1. Schematic illustration of the symmetry of the silicon crystal plane and optomechanical crystal.

The symmetry group of the stiffness tensor of silicon in the crystal plane and the hexagonal optomechanical crystal is C_{4v} and C_{6v}, respectively. For both C_{4v} and C_{6v} groups, there are two sets of equivalent mirror planes σ_v and σ_d as shown in Fig. S1. When one mirror plane of the silicon crystal lattice aligns with one mirror plane of the hexagonal optomechanical crystal, i.e., $\theta = 0°$, 15°, 30°, and 45°, the mechanical system restores mirror symmetry and is described by C_{2v} group. Otherwise, the symmetry group for the mechanical system is C_2.

The far-field acoustic plane wave propagating along the $z-$direction can be written as

$$\boldsymbol{Q}_0 = \boldsymbol{Q}_{T,0} + \boldsymbol{Q}_{L,0} = (u\boldsymbol{e}_x + v\boldsymbol{e}_y)\, e^{ik^0_{T,z}z} + w\boldsymbol{e}_z e^{ik^0_{L,z}z}. \tag{S1}$$

According to Tables S1 and S2, $\boldsymbol{e}_x$ and $\boldsymbol{e}_y$ belong to B_1 and B_2 representations of C_{2v} and B representation of C_2, respectively, and $\boldsymbol{e}_z$ belongs to A_1 representation of C_{2v} and A representation of C_2. Therefore, a mechanical mode at the Γ point can be a BIC, i.e., decouples from both transverse and longitudinal radiation waves, only if it belongs to the A_2 representation of C_{2v} group when $\theta = 0°$, 15°, 30°, or 45°.

TABLE S1. Character table of the C_{2v} point group

C_{2v}	E	C_2	σ_v	σ_d
A_1	1	1	1	1
A_2	1	1	-1	-1
B_1	1	-1	1	-1
B_2	1	-1	-1	1

* These authors contributed equally to this work.
† kfang3@illinois.edu

TABLE S2. Character table of the C_2 point group

C_2	E	C_2
A	1	1
B	1	-1

Next we analyze the optomechanical coupling of a unit cell based on the symmetry. The unit-cell optomechanical coupling is given by

$$g = g_{\mathrm{MB}} + g_{\mathrm{PE}} \equiv \sqrt{\frac{\hbar}{2m_{\mathrm{eff}}\omega_m}}\,(g_{\mathrm{OM,MB}} + g_{\mathrm{OM,PE}}) \tag{S2}$$

where $m_{\mathrm{eff}} = \int_{\mathrm{u.c.}} \rho|\boldsymbol{Q}|^2 \; \mathrm{d}V$ is the unit-cell effective mass, ω_m is the mechanical frequency. The moving-boundary and photoelastic components are calculated as

$$g_{\mathrm{OM,MB}} = -\frac{\omega_o}{2}\frac{\int_{\mathrm{u.c.}} (\boldsymbol{Q}\cdot\hat{\boldsymbol{n}})\left(\Delta\epsilon\left|\boldsymbol{E}_{\parallel}\right|^2 - \Delta\epsilon^{-1}\left|\boldsymbol{D}_{\perp}\right|^2\right)\; \mathrm{d}S}{\int_{\mathrm{u.c.}} \boldsymbol{E}^*\cdot\boldsymbol{D}\; \mathrm{d}V}, \tag{S3}$$

and

$$g_{\mathrm{OM,PE}} = \frac{\omega_o}{2}\frac{\int_{\mathrm{u.c.}} \epsilon_0 n^4 E_i^* E_j p_{ijkl} S_{kl}\; \mathrm{d}V}{\int_{\mathrm{u.c.}} \boldsymbol{E}^*\cdot\boldsymbol{D}\; \mathrm{d}V}, \tag{S4}$$

where ω_o is the optical frequency, $\hat{\boldsymbol{n}}$ is the normal vector of the interfaces, $\boldsymbol{Q}$ is displacement, S_{kl} is strain tensor, the subscripts $\parallel$ and $\perp$ indicate the field components parallel and perpendicular to the surface and $\Delta\epsilon = \epsilon_{\mathrm{int}} - \epsilon_{\mathrm{ext}}$ and $\Delta\epsilon^{-1} = \epsilon_{\mathrm{int}}^{-1} - \epsilon_{\mathrm{ext}}^{-1}$ (ϵ_{ext} is the permittivity of the media which $\hat{\boldsymbol{n}}$ points to and ϵ_{int} is the permittivity of the media on the other side).

For the moving boundary term, we write the numerator using a shorthanded notation $\int_{\mathrm{u.c.}} (\boldsymbol{Q}\cdot\hat{\boldsymbol{n}}) f\; \mathrm{d}S$. When there is mirror symmetry, this integration can be calculated as

$$\begin{aligned}
\int_{\mathrm{u.c.}} \boldsymbol{Q}\cdot\hat{\boldsymbol{n}} f\; \mathrm{d}S &= \left(\int_{\mathrm{u.c.}} \boldsymbol{Q}(\boldsymbol{r})\cdot\hat{\boldsymbol{n}}(\boldsymbol{r}) f(\boldsymbol{r})\; \mathrm{d}S + \int_{\mathrm{u.c.}} \boldsymbol{Q}(\sigma\boldsymbol{r})\cdot\hat{\boldsymbol{n}}(\sigma\boldsymbol{r}) f(\sigma\boldsymbol{r})\; \mathrm{d}S\right)/2 \\
&= \left(\int_{\mathrm{u.c.}} \boldsymbol{Q}(\boldsymbol{r})\cdot\hat{\boldsymbol{n}}(\boldsymbol{r}) f(\boldsymbol{r})\; \mathrm{d}S + \int_{\mathrm{u.c.}} \sigma^{-1}\boldsymbol{Q}(\boldsymbol{r})\cdot\hat{\boldsymbol{n}}(\boldsymbol{r})\sigma^{-1} f(\boldsymbol{r})\; \mathrm{d}S\right)/2 \\
&= (1+\chi_{\boldsymbol{Q}}(\sigma))\int_{\mathrm{u.c.}} \boldsymbol{Q}(\boldsymbol{r})\cdot\hat{\boldsymbol{n}}(\boldsymbol{r}) f(\boldsymbol{r})\; \mathrm{d}S/2,
\end{aligned} \tag{S5}$$

where $\chi_{\boldsymbol{Q}}(\sigma)$ stands for the character of mirror symmetry operation σ. Thus, the integral vanishes when the mechanical mode is odd with respect to the mirror plane. We have already used the fact that $f(\boldsymbol{r})$ is even under the mirror operation as it is a quadratic function of $\boldsymbol{E}$ and $\boldsymbol{D}$. This derivation is valid only when both mechanical and optical modes share the same mirror plane, which is true for $\theta = 0°$ and $\theta = 45°$. For $\theta = 15°$ and $\theta = 30°$, $g_{\mathrm{OM,MB}}$ in general will be nonzero.

To calculate the photoelastic term, we note the photoelastic tensor for cubic crystal, such as silicon, in Voigt notation is given by

$$p_{\mathrm{C}} = \begin{pmatrix} p_{11} & p_{12} & p_{12} & & & \\ & p_{11} & p_{12} & & & \\ & & p_{11} & & & \\ & & & p_{44} & & \\ & \text{symm.} & & & p_{44} & \\ & & & & & p_{44} \end{pmatrix}. \tag{S6}$$

We perform the computation in the frame of optomechanical crystal by rotating the silicon crystal lattice. Under a rotating $R(\theta)$, the photoelastic tensor transforms as $p'_{ijkl}(\theta) = R_{ip}(\theta)R_{jq}(\theta)R_{kr}(\theta)R_{ls}(\theta)p_{pqrs}$, or in the Voigt notation,

$$\begin{gathered}
p'_{11} = p'_{22} = \frac{1}{4}\left[p_{11}(\cos 4\theta + 3) + (p_{12} + 2p_{44})(1 - \cos 4\theta)\right] \\
p'_{33} = p_{33} \\
p'_{12} = p'_{21} = \frac{1}{4}\left[p_{12}(\cos 4\theta + 3) + (p_{11} - 2p_{44})(1 - \cos 4\theta)\right] \\
p'_{13} = p'_{23} = p'_{31} = p'_{32} = p_{12} \\
p'_{44} = p'_{55} = p_{44} \\
p'_{66} = \frac{1}{4}\left[2p_{44}(\cos 4\theta + 1) + (p_{11} - p_{12})(1 - \cos 4\theta)\right] \\
p'_{16} = p'_{61} = \frac{1}{4}\sin 4\theta\,(p_{11} - p_{12} - 2p_{44}) \\
p'_{26} = p'_{62} = -p'_{16}.
\end{gathered} \tag{S7}$$

The integral in the photoelastic coupling term is expressed as

$$\int \epsilon_0 n^4 E_i^* E_j p_{ijkl} S_{kl}\,\mathrm{d}V = \int \epsilon_0 n^4 (p'_{11} f_{11} + p'_{12} f_{12} + p'_{13} f_{13} + p'_{31} f_{31} + p'_{33} f_{33} + p'_{44} f_{44} + p'_{66} f_{66} + p'_{16} f_{16})\,\mathrm{d}V, \tag{S8}$$

where

$$\begin{gathered}
f_{11} = |E_x|^2 S_{xx} + |E_y|^2 S_{yy} \\
f_{12} = |E_x|^2 S_{yy} + |E_y|^2 S_{xx} \\
f_{13} = |E_x|^2 S_{zz} + |E_y|^2 S_{zz} \\
f_{31} = |E_z|^2 S_{xx} + |E_z|^2 S_{yy} \\
f_{33} = |E_z|^2 S_{zz} \\
f_{44} = 2\mathrm{Re}\left\{E_y^* E_z\right\} \cdot 2S_{yz} + 2\mathrm{Re}\left\{E_x^* E_z\right\} \cdot 2S_{xz} \\
f_{16} = \left(|E_x|^2 - |E_y|^2\right) \cdot 2S_{xy} + 2\mathrm{Re}\left\{E_x^* E_y\right\} \cdot (S_{xx} - S_{yy}).
\end{gathered} \tag{S9}$$

When $\theta = 0°$ and $45°$, there are mirror planes σ_x and σ_y. We list the symmetry of $E_i^* E_j$, S_{kl} and f_{ij} in Tables I and I, where 'e' stands for even and 'o' stands for odd.

		$\|E_x\|^2$	$\|E_y\|^2$	$\|E_z\|^2$	$\mathrm{Re}\{E_x^* E_y\}$	$\mathrm{Re}\{E_x^* E_z\}$	$\mathrm{Re}\{E_y^* E_z\}$
$\chi(\sigma_x) = \pm 1$	under σ_x	e	e	e	o	o	e
$\chi(\sigma_y) = \pm 1$	under σ_y	e	e	e	o	e	o
		S_{xx}	S_{yy}	S_{zz}	S_{xy}	S_{xz}	S_{yz}
$\chi(\sigma_x) = 1$	under σ_x	e	e	e	o	o	e
$\chi(\sigma_x) = -1$	under σ_x	o	o	o	e	e	o
$\chi(\sigma_y) = 1$	under σ_y	e	e	e	o	e	o
$\chi(\sigma_y) = -1$	under σ_y	o	o	o	e	o	e

TABLE S3. The behavior of $E_i^* E_j$ and S_{ij} under σ_x and σ_y.

We find that if the mechanical mode is odd under either σ_x or σ_y, all the f_{ij}s except f_{16} are odd and their integrals are zero. For f_{16}, because $p'_{16} = 0$ when $\theta = 0°$ or $45°$, the term $\int p'_{16} f_{16}\,\mathrm{d}V$ also vanishes. For $\theta = 15°$ and $30°$, $g_{\mathrm{OM,PE}}$ is in general nonzero.

In summary, A_2 modes for $\theta = 0°$ and $45°$, which are BICs, have a zero optomechanical coupling. For other cases, optomechanical coupling is in general nonzero. This is also summarized in Table I of the main text.

		f_{11}	f_{12}	f_{13}	f_{31}	f_{33}	f_{44}	f_{66}	f_{16}
$\chi(\sigma_x) = 1$	under σ_x	e	e	e	e	e	e	e	o
$\chi(\sigma_x) = -1$	under σ_x	o	o	o	o	o	o	o	e
$\chi(\sigma_y) = 1$	under σ_y	e	e	e	e	e	e	e	o
$\chi(\sigma_y) = -1$	under σ_y	o	o	o	o	o	o	o	e

TABLE S4. The behavior of f_{ij} under σ_x and σ_y.

II. OPTOMECHANICAL COUPLING OF FINITE OPTOMECHANICAL CRYSTALS

The optomechanical coupling for a sufficiently large optomechanical crystal can be calculated with the result of the unit-cell coupling. Performing the integrals of Eqs. S3 and S4 in the whole optomechanical crystal and apply the Bloch theorem, i.e., $\boldsymbol{Q}(\boldsymbol{r}+\boldsymbol{R}) = e^{i\boldsymbol{k}_m\cdot\boldsymbol{R}}\boldsymbol{Q}(\boldsymbol{r})$ and $\boldsymbol{E}(\boldsymbol{r}+\boldsymbol{R}) = e^{i\boldsymbol{k}_o\cdot\boldsymbol{R}}\boldsymbol{E}(\boldsymbol{r})$, where $\boldsymbol{k}_m$ and $\boldsymbol{k}_o$ are the Bloch wavevector of the mechanical and optical modes, respectively, we have

$$\bar{g}_{\mathrm{OM,MB}} = -\frac{\omega_o}{2}\frac{\sum\limits_{\boldsymbol{R}} e^{i\boldsymbol{k}_m\cdot\boldsymbol{R}}\int_{\mathrm{u.c.}}(\boldsymbol{Q}\cdot\hat{\boldsymbol{n}})(\Delta\epsilon\left|\boldsymbol{E}_\parallel\right|^2 - \Delta\epsilon^{-1}|\boldsymbol{D}_\perp|^2)\,\mathrm{d}S}{\sum\limits_{\boldsymbol{R}}\int_{\mathrm{u.c.}}\boldsymbol{E}^*\cdot\boldsymbol{D}\,\mathrm{d}V} = \frac{\sum\limits_{\boldsymbol{R}} e^{i\boldsymbol{k}_m\cdot\boldsymbol{R}}}{N_c}g_{\mathrm{OM,MB}} \tag{S10}$$

where N_c is the number of unit cells and $\sum\limits_{\boldsymbol{R}}$ is the summation for all the lattice vectors. Same expression holds for $g_{\mathrm{OM,PE}}$. Because $\sum\limits_{\boldsymbol{R}} e^{i\boldsymbol{k}_m\cdot\boldsymbol{R}}$ is nonzero only when $\boldsymbol{k}_m = 0$, optomechanical coupling exists only for mechanical modes at the Γ point. Because the total mass $\bar{m}_{\mathrm{eff}} = N_c m_{\mathrm{eff}}$, the optomechanical coupling of a sufficiently large optomechanical crystal is related to its unit-cell coupling as

$$\bar{g} = \sqrt{\frac{\hbar}{2N_c m_{\mathrm{eff}}\omega_m}}(g_{\mathrm{OM,MB}} + g_{\mathrm{OM,PE}}) = \frac{g}{\sqrt{N_c}}. \tag{S11}$$

For finite optomechanical crystals, we have to consider the field envelop of the standing-wave resonances. The fields across unit cells now are related by $\boldsymbol{Q}(\boldsymbol{r}+\boldsymbol{R}) = e^{i\boldsymbol{k}_m\cdot\boldsymbol{R}}\phi_{(p_m,q_m)}(\boldsymbol{R})\boldsymbol{Q}(\boldsymbol{r})$ and $\boldsymbol{E}(\boldsymbol{r}+\boldsymbol{R}) = e^{i\boldsymbol{k}_o\cdot\boldsymbol{R}}\phi_{(p_o,q_o)}(\boldsymbol{R})\boldsymbol{E}(\boldsymbol{r})$, where $\phi_{(p,q)}(\boldsymbol{R})$ is the envelop function of the (p,q)-th order standing-wave resonance. The envelope function can be approximately solved as the eigenfunction of a flat-top potential well within the boundary of the finite optomechanical crystal. Then we have

$$\begin{aligned}\bar{g}_{\mathrm{OM,MB}} &= -\frac{\omega_o}{2}\frac{\sum\limits_{\boldsymbol{R}} e^{i\boldsymbol{k}_m\cdot\boldsymbol{R}}\phi_{(p_m,q_m)}(\boldsymbol{R})\left|\phi_{(p_o,q_o)}(\boldsymbol{R})\right|^2\int_{\mathrm{u.c.}}(\boldsymbol{Q}\cdot\hat{\boldsymbol{n}})(\Delta\epsilon\left|\boldsymbol{E}_\parallel\right|^2 - \Delta\epsilon^{-1}|\boldsymbol{D}_\perp|^2)\,\mathrm{d}S}{\sum\limits_{\boldsymbol{R}}\left|\phi_{(p,q)}(\boldsymbol{R})\right|^2\int_{\mathrm{u.c.}}\boldsymbol{E}^*\cdot\boldsymbol{D}\,\mathrm{d}V} \\ &= \frac{\sum\limits_{\boldsymbol{R}} e^{i\boldsymbol{k}_m\cdot\boldsymbol{R}}\phi_{(p_m,q_m)}(\boldsymbol{R})\left|\phi_{(p_o,q_o)}(\boldsymbol{R})\right|^2}{\sum\limits_{\boldsymbol{R}}\left|\phi_{(p_o,q_o)}(\boldsymbol{R})\right|^2}g_{\mathrm{OM,MB}} \\ &\approx \frac{\int e^{i\boldsymbol{k}_m\cdot\boldsymbol{R}}\phi_{(p_m,q_m)}(\boldsymbol{R})\left|\phi_{(p_o,q_o)}(\boldsymbol{R})\right|^2\,\mathrm{d}S}{\int\left|\phi_{(p_o,q_o)}(\boldsymbol{R})\right|^2\,\mathrm{d}S}g_{\mathrm{OM,MB}}.\end{aligned} \tag{S12}$$

where the last integrals are performed in the whole optomechanical crystal. Similar expression applies to the photoelastic term. For the total effective mass, we have

$$\bar{m}_{\mathrm{eff}} = \sum_{\boldsymbol{R}}\int_{\mathrm{u.c.}}\rho|\boldsymbol{Q}|^2\,\mathrm{d}V = \sum_{\boldsymbol{R}}\left|\phi_{(p_m,q_m)}(\boldsymbol{R})\right|^2\int_{\mathrm{u.c.}}\rho|\boldsymbol{Q}|^2\,\mathrm{d}V = \sum_{\boldsymbol{R}}\left|\phi_{(p_m,q_m)}(\boldsymbol{R})\right|^2 m_{\mathrm{eff}} \approx N_c\frac{\int\left|\phi_{(p_m,q_m)}(\boldsymbol{R})\right|^2\,\mathrm{d}S}{\int\mathrm{d}S}m_{\mathrm{eff}}. \tag{S13}$$

Finally, the optomechanical coupling of a finite optomechanical crystal is related to its unit-cell coupling by

$$\bar{g} = \frac{\zeta g}{\sqrt{N_c}}, \tag{S14}$$

where the finite-size correction factor ζ is given by

$$\zeta = \frac{\int e^{i\boldsymbol{k}_m \cdot \boldsymbol{R}} \phi_{(p_m,q_m)}(\boldsymbol{R}) \left|\phi_{(p_o,q_o)}(\boldsymbol{R})\right|^2 \mathrm{d}S}{\int \left|\phi_{(p_o,q_o)}(\boldsymbol{R})\right|^2 \mathrm{d}S \sqrt{\int \left|\phi_{(p_m,q_m)}(\boldsymbol{R})\right|^2 \mathrm{d}S / \int \mathrm{d}S}}. \tag{S15}$$

For the mechanical Bloch wavevector $\boldsymbol{k}_m = 0$, the numerically-calculated correction factor for a few standing-wave mechanical and optical resonances of a hexagonal crystal is listed in Table S5. The field envelops of some orders are shown in Fig. 3c. The order of the hexagonal crystal mode can be hard to identify. We determined it by adiabatically deforming the hexagonal crystal to a rectangular crystal and tracing the corresponding mode, where (p, q)-th resonance has $p - 1$ and $q - 1$ nodes along the x and y direction, respectively.

TABLE S5. Correction factor ζ for some standing-wave mechanical and optical resonances of a hexagonal crystal.

Mechanical \ Optical	(1,1)	(1,2)	(2,1)	(2,2)	(3,1)
(1,1)	1.4075	1.1427	1.1415	0.9737	0.9740
(3,1)	0.0185	0.8250	0.8322	0.0183	0.0805
(1,3)	0.3987	0.4442	0.3692	0.7307	0.7398

The interaction Hamiltonian of the finite optomechanical crystal involving a pair of mechanical and optical standing-wave resonances is given by $\hat{H} = \bar{g}\hat{a}^\dagger\hat{a}(\hat{b}^\dagger + \hat{b})$, where $\hat{a}^\dagger(\hat{a})$ and $\hat{b}^\dagger(\hat{b})$ are the creation(annihilation) operators of the optical and mechanical resonances, respectively. When the optomechanical crystal is driven by a pump detuned from the optical resonance by the mechanical frequency, the interaction Hamiltonian could be linearized to be $\hat{H} = G(\hat{a}^\dagger\hat{b}^\dagger + \hat{a}\hat{b})$ for blue-detuned pump or $\hat{H} = G(\hat{a}^\dagger\hat{b} + \hat{a}\hat{b}^\dagger)$ for red-detuned pump, with

$$G = \bar{g}\sqrt{n} = \zeta g\sqrt{n_c} \tag{S16}$$

the parametrically-enhanced optomechanical coupling, where n and $n_c = n/N_c$ are the total photon number in the optical resonance and average photon number per unit cell, respectively.

III. MECHANICAL NOISE POWER SPECTRUM

The total noise power spectral density S measured by the photodetector is given by [1, 2]

$$S = S_e + \frac{G_e^2}{R_I}\left(S_{\mathrm{EDFA}} + G_{\mathrm{EDFA}}^2 S_{\mathrm{SN}}^2\left(1 + \eta\frac{\kappa_e}{\kappa}\frac{8G^2}{\kappa}S_m[\omega]\right)\right), \tag{S17}$$

where S_e is the electronic noise of the detector, S_{EDFA} is the noise of EDFA, $S_{\mathrm{SN}} = \sqrt{P_{\mathrm{out}}\hbar\omega_l}$ is the optical shot noise,, $S_m[\omega]$ is the mechanical noise spectrum, $\kappa = \kappa_i + \kappa_e$ is the total optical dissipation, G is the parametrically-enhanced optomechanical coupling, η is the total detection efficiency, G_e is the detector gain factor from optical power to voltage, G_{EDFA} is the EDFA gain, and R_I is the input impedance of the spectrum analyzer. The optically-transduced mechanical noise spectrum is given by

$$S_m[\omega] = \frac{1}{2}\left(\frac{\gamma(\bar{n}_m + 1)}{(\omega_m - \omega)^2 + (\gamma/2)^2} + \frac{\gamma\bar{n}_m}{(\omega_m + \omega)^2 + (\gamma/2)^2}\right) \tag{S18}$$

where $\bar{n}_m = \frac{k_B T}{\hbar\omega_m}$ the thermal occupation of the mechanical mode. The total detection efficiency is $\eta = \eta_{\mathrm{cpl}}\eta_{\mathrm{t}}\eta_{\mathrm{det}}$, where η_{cpl} is the coupling efficiency of the grating coupler at the pump wavelength, η_{t} is the total transmission

efficiency in the optical fiber path, and $\eta_{\rm det}$ is the quantum efficiency of the photodetector. We measured $\eta_{\rm t} = 0.80$ and $\eta_{\rm cpl} \approx 0.5$ depending on the pump wavelength, while $\eta_{\rm det} = 0.68$ is given by the detector, which gives $\eta \approx 0.27$.

We normalize the noise power spectrum with respect to the total noise floor which yields

$$\overline{S} = 1 + \frac{S'_{\rm SN}}{S_e + S'_{\rm EDFA} + S'_{\rm SN}} \eta \frac{\kappa_e}{\kappa} \frac{8G^2}{\kappa} S_m[\omega], \tag{S19}$$

where we have used $S'_{\rm EDFA} \equiv \frac{G_e^2}{R_I} S_{\rm EDFA}$ and $S'_{\rm SN} \equiv \frac{G_e^2 G_{\rm EDFA}}{R_I} S_{\rm SN}$ to denote the photodetector-measured EDFA noise and laser shot noise. The electronic noise S_e is measured by blocking all the light. $S'_{\rm SN} + S_e$ can be determined by removing the EDFA while keeping the laser power incident to the detector unchanged. Thus we obtain $S'_{\rm SN}$ and the ratio of $\frac{S'_{\rm SN}}{S_e + S'_{\rm EDFA} + S'_{\rm SN}}$. Finally, the measured normalized noise power spectrum is fitted using Eqs. S19 and S18, with ω_m, γ, and G the only fitting parameters. The unit-cell optomechanical coupling is calculated from $g = G/(\zeta\sqrt{n_c})$, using $n_c = \kappa_e P/((\omega_m)^2 + (\kappa/2)^2)/\frac{3\sqrt{3}}{2} N^2$ and ζ depending on the optical standing-wave resonance that is deployed (see Table S5). For the four devices shown in Fig. 4a as an example, we used optical resonances of the following order, 0° (1,1), 15° (1,1), 30° (1,1), 45° (2,1), while the observed mechanical resonances are (1,1).

IV. FABRICATION RESULTS

The silicon-on-insulator microchip (220 nm silicon device layer and 3 μm buried oxide layer) is first rinsed with Acetone, isopropyl alcohol and deionized water to clean the surface. The pattern is then defined using electron beam lithography (Elionix ELS-G150) with 300 nm thick ZEP520A mask, followed by inductively coupled plasma reactive ion etch (Oxford PlasmaPro 100 Cobra) of silicon using SF6 and CHF3. The mask residual is removed by n-methyl-2-pyrrolidone hot bath and oxygen plasma cleaning (Diener Descum).

The SEM image of a typical unit cell is shown in Fig.S2a. The disorder of the snowflake patterns are measured both inside a same optomechanical crystal and across different optomechanical crystals (Table S6). The standard deviation of r and w is about 2.7% and 7.22% for the former and 2.6% and 6.7% for the latter. For comparison of different optomechanical crystals, we examined unit cells from the same location. Roughly speaking, the variation across different devices accounts for the the resonance frequency fluctuation and disorder within an optomechanical crystal induces scattering loss and decrease the resonance quality factor (see Section V).

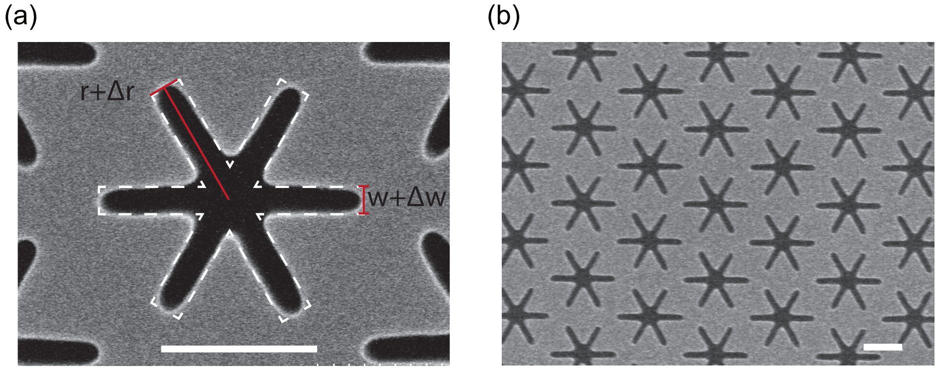

FIG. S2. **a**. An SEM image of a typical unit cell. The dashed line shows the fitted snowflake structure. **b**. An SEM image of a few unit cells within an optomechanical crystal. The white bar refers to 200 nm for both images.

V. SIMULATION OF SCATTERING LOSS

In multimode photonic/phononic crystals, structural disorders could cause scattering among different modes and thus introduce more loss channels to a given mode [3]. To estimate the quality factor of standing-wave mechanical resonances in disordered crystals, we simulated a 1×4 super-cell structure (Fig. S3a), where variations of both

TABLE S6. Mean and standard deviation of r and w.

	Across different devices	Inside one device
$\bar{r}$ (nm)	172.09	167.35
$r_{\max}$ (nm)	177.60	174.65
$r_{\min}$ (nm)	161.00	154.65
Δr (nm)	4.49	4.57
$\bar{w}$ (nm)	37.76	37.26
$w_{\max}$ (nm)	44.00	43.10
$w_{\min}$ (nm)	31.70	32.70
Δw (nm)	2.53	2.67

length (Δr) and width (Δw) of each snowflake hole are introduced. Each Δr and Δw are independently sampled and the standard deviation of Δr and Δw are controlled to be 4 nm and 2 nm, respectively, in accordance with the fabricated devices. We run 50 sets of simulations each for $\theta = 0°$, $15°$, $30°$, and $45°$. We set the Bloch wavevector to be $k = 2\pi/(3aN)$ with $N = 25$, approximately corresponding to the $(1, 1)$ standing-wave resonance in size $N = 25$ hexagonal optomechanical crystals. The simulated radiative quality factors Q_r combined with room-temperature material absorption Q_a, which we assumed to be 3000, gives the total mechanical quality factor: $1/Q_m = 1/Q_r + 1/Q_a$. The result is plotted in Fig. S3b. Due to the small size of the optomechanical crystal, there are no significant variations of Q_m among four different θ's. We use the data set of all four angles here to calculate the upper and lower bounds of Q_m as shown in Fig. 4.

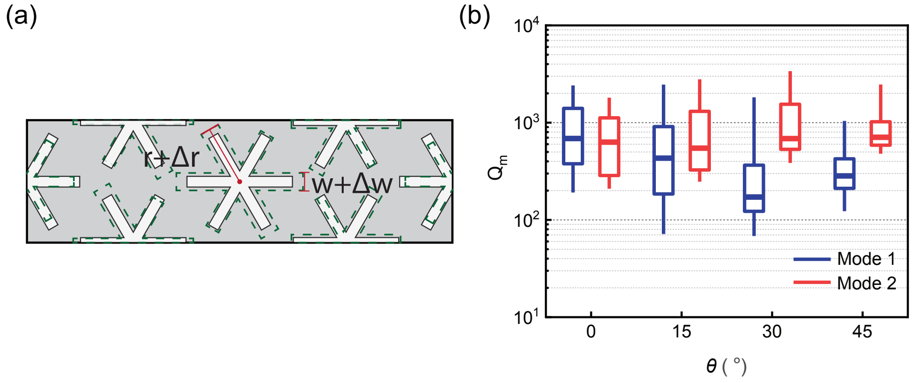

FIG. S3. **a**. A schematic diagram of a 1×4 super-cell structure with disorders Δr and Δw. **b**. The simulated Q_m for different θ. The lower and upper edges of the boxes represent the 16% and 84% bounds of the data, respectively, and the middle lines is the median value.

VI. DESIGN OF GRATING COUPLER

Fig. S4a shows the fiber-optic coupler for the measurement of the optomechanical crystal. Light is guided by an angle-polished single-mode fiber, reflects at the polished surface, and is incident onto a grating coupler. The angle α of the fiber is chosen to ensure total internal reflection. Because of the inevitable air gap between the fiber and grating coupler, the incident angle onto the grating coupler is given by $\beta = \arcsin(n_{\text{fiber}} \cdot \sin(90° - 2\alpha))$. In general, the grating coupler is designed to convert the incident light to a guided wave by satisfying the phase-matching condition $\frac{2\pi n_{\text{eff}}}{\lambda} = \frac{2\pi}{\lambda} \sin\beta + \frac{2\pi}{\Lambda}$, where n_{eff} is the effective refractive index of the grating and Λ is the lattice constant of the grating. It turns out an apodized grating coupler could yield higher coupling efficiency than a uniform grating coupler because of the better mode-matching with the single-mode optical fiber [4]. Thus, we adopt the apodized grating

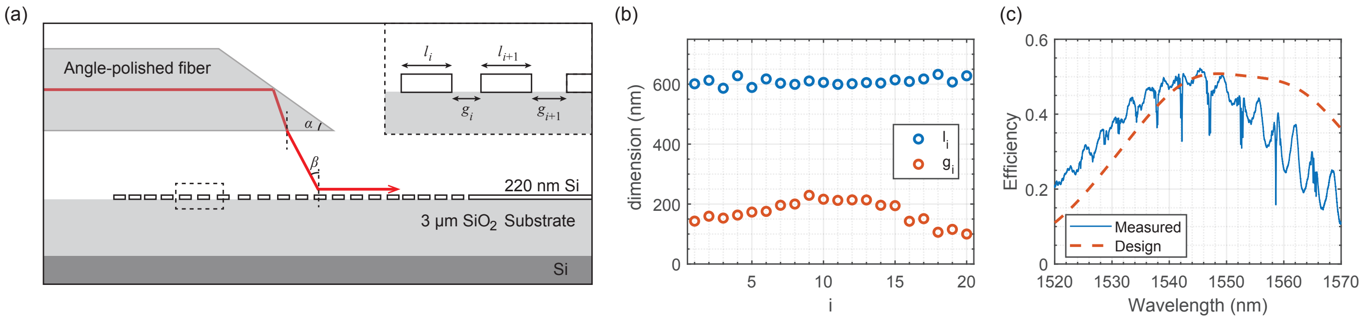

FIG. S4. **a**. Schematic plot of the angle-polished fiber and grating coupler. **b**. Parameters l_i and g_i for the optimized grating coupler. **c**. Measured and simulated efficiency of the optimized grating coupler.

coupler scheme. The apodized grating coupler consists of 20 unit-cells with 40 individual tunable parameters l_i and g_i. The parameters are optimized using a standard inverse design approach [5] to maximize the coupling efficiency for different fiber angles. The optimized fiber angle is found to be 35.5° and the corresponding l_i and g_i are shown in Fig. S4b. Fig. S4c shows the simulated transmission and measured device spectrum (inferred from the reflection spectrum). The peak efficiency is observed to be 52% with a 3-dB bandwidth of 40 nm.

VII. DESIGN OF PHOTONIC CRYSTAL MIRROR

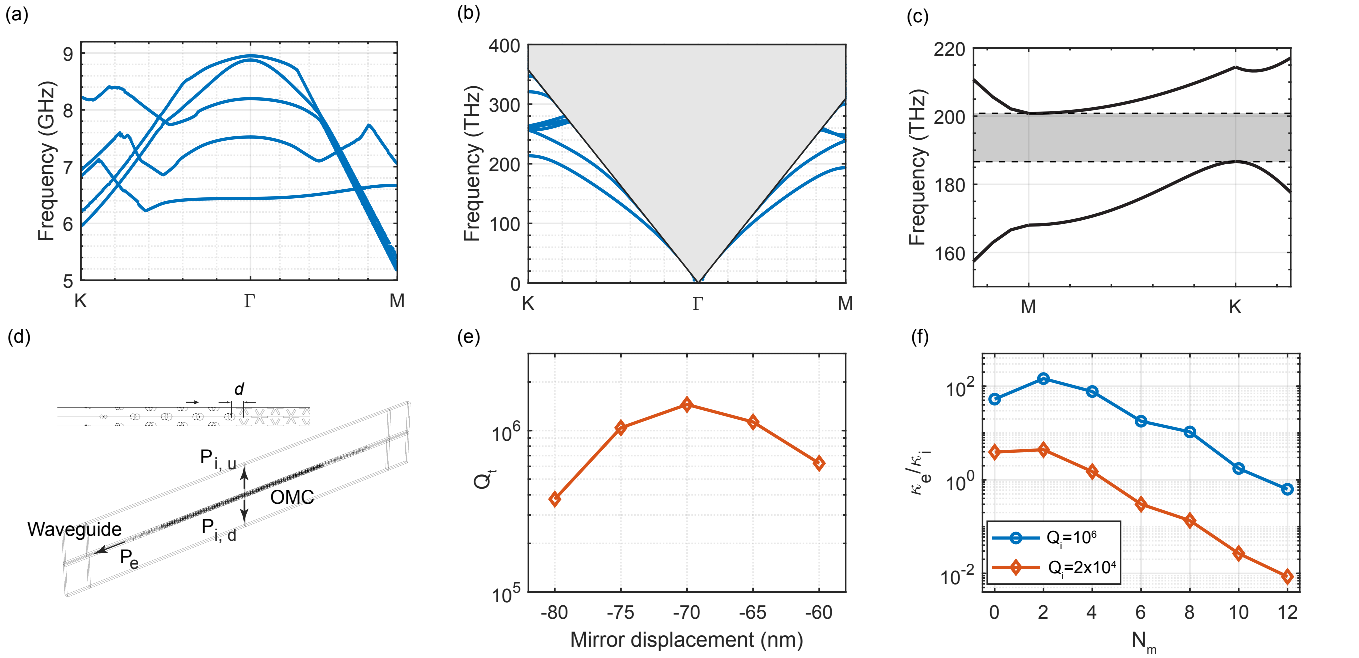

FIG. S5. **a** and **b** Mechanical and optical bandstructure of the nominal optomechanical crystal. **c**. Band structure of the photonic crystal mirror. **d**. Super-cell used in the simulation. Inset: Top view of the junction between the optomechanical crystal and waveguide. Dashed circles are the original location of the photonic crystal mirror holes before displacement. **e**. Simulated total quality factor Q_t versus the displacement $d-a$. **f.** Simulated κ_e/κ_i versus the number of nominal mirror layers N_m for $Q_i = 10^6$ and $Q_i = 2 \times 10^4$.

Fig. S5a and b show the full mechanical and optical bandstructure of the nominal optomechanical crystal. The photonic crystal mirror consists of a triangular lattice of cylindrical holes with $r = 78$ nm and the same lattice constant $a = 389$ nm as the snowflake optomechanical crystal. The simulated band structure with the two lowest TE-like bands (Fig. S5c) shows a complete bandgap from 187 THz (1606 nm) to 201 THz (1493 nm). The photonic crystal mirror effectively suppresses the side-leakage but also perturbs the tail of the standing-wave resonance, inducing wavevector

components that fall inside the continuum and causing out-of-plane radiation. We could alleviate such out-of-plane radiation by tuning the separation d between the photonic crystal mirror and optomechanical crystal [6, 7]. To simulate this, we take a strip of optomechanical crystal of 25 unit cells as one super-cell and add periodic boundary conditions on the transverse direction, as shown in Fig. S5d. A sufficient number of photonic crystal mirror unit-cells are included and so the total quality factor Q_t of the resonance is dominated by the out-of-plane radiation. Fig. S5e shows $Q_t \equiv \frac{\mathrm{Re}[\omega_o]}{\mathrm{Im}[\omega_o]}$ of the fundamental standing-wave resonance for different d. An optimal $d = 319$ nm is observed, corresponding to a displacement of the photonic crystal from the nominal lattice constant by $d - a = -70$ nm.

The fabricated optomechanical crystal connects to a waveguide and the external coupling rate could be controlled by the number of phtonic crystal mirror layers between the waveguide and optomechanical crystal. In the simulation, we change the number of phtonic crystal mirror layers N_m on one side (the waveguide side) of the optomechanical crystal super-cell while keeping the other side with a sufficient amount of photonic crystal mirror layers. We have also included four additional tapered mirror layers on the waveguide side. The external and internal quality factors are inferred from the energy flux as

$$Q_e = \frac{P_e + P_{i,u} + P_{i,d}}{P_e} Q_t, \quad Q_i = \frac{P_e + P_{i,u} + P_{i,d}}{P_{i,u} + P_{i,d}} Q_t, \tag{S20}$$

where P_e is the energy flux towards the waveguide and $P_{i,u}$ and $P_{i,d}$ are out-of-plane energy flux upward and downward, respectively. Fig. S5f shows $\kappa_e/\kappa_i \equiv Q_i/Q_e$. The simulation is done for two cases. One case is with a large number of photonic crystal mirrors on the other side of the super-cell such that $Q_i \approx 10^6$. In the other case, we purposely reduced the number of photonic crystal mirrors on the other side of the super-cell such that $Q_i \approx 2 \times 10^4$ to mimic the fabricated optomechanical crystal. For this case. We find the critical coupling $\kappa_e/\kappa_i \approx 1$ is achieved around $N_m = 4$.

[1] Meenehan, S. M., Cohen, J. D., Gröblacher, S., Hill, J. T., Safavi-Naeini, A. H., Aspelmeyer, M. & Painter, O. Silicon optomechanical crystal resonator at millikelvin temperatures. *Physical Review A* **90**, 011803 (2014).

[2] Safavi-Naeini, A. H., Chan, J., Hill, J. T., Gröblacher, S., Miao, H., Chen, Y., Aspelmeyer, M. & Painter, O. Laser noise in cavity-optomechanical cooling and thermometry. *New Journal of Physics* **15**, 035007 (2013).

[3] Jin, J., Yin, X., Ni, L., Soljačić, M., Zhen, B. & Peng, C. Topologically enabled ultrahigh-q guided resonances robust to out-of-plane scattering. *Nature* **574**, 501–504 (2019).

[4] Zhao, Z. & Fan, S. Design principles of apodized grating couplers. *Journal of Lightwave Technology* **38**, 4435–4446 (2020).

[5] Molesky, S., Lin, Z., Piggott, A. Y., Jin, W., Vucković, J. & Rodriguez, A. W. Inverse design in nanophotonics. *Nature Photonics* **12**, 659–670 (2018).

[6] Akahane, Y., Asano, T., Song, B.-S. & Noda, S. Fine-tuned high-Q photonic-crystal nanocavity. *Optics Express* **13**, 1202–1214 (2005).

[7] Chen, Z., Yin, X., Jin, J., Zheng, Z., Zhang, Z., Wang, F., He, L., Zhen, B. & Peng, C. Observation of miniaturized bound states in the continuum with ultra-high quality factors. *Science Bulletin* **67**, 359–366 (2022).
